# Phenolic Indices and Antioxidant Properties of a Thermally Processed Model Matrix Containing Dried *Cynanchum thesioides* (Freyn) K. Schum Powder

**DOI:** 10.3390/molecules31152603

**Published:** 2026-07-25

**Authors:** Tao Lyu, Yuchen Cheng, Woonjung Kim

**Affiliations:** 1 Department of Nanomaterials and Semiconductor, Hannam University, Daejeon 34430, Republic of Korea; lyutao@naver.com; 2 Department of Chemistry, Hannam University, Daejeon 34430, Republic of Korea

**Keywords:** cookie fortification, radical scavenging, ferric ion reduction, Maillard reaction, functional ingredient, wheat flour replacement

## Abstract

Phenolic compounds are valued for their antioxidant activity, but their measured response after heating can vary with the surrounding matrix. In this study, dried *Cynanchum thesioides* (Freyn) K. Schum powder (CTP) was added to a cookie matrix at 0%, 1%, 3%, and 5% (*w*/*w*) to examine phenolic indices, antioxidant activity, and physicochemical changes after baking. CTP affected moisture content, spread ratio, hardness, color, pH, and soluble solids in the baked matrix, whereas density was unchanged. Antioxidant activity increased with increasing CTP level. The 2,2-diphenyl-1-picrylhydrazyl (DPPH) and 2,2′-azino-bis(3-ethylbenzothiazoline-6-sulfonic acid) (ABTS) radical scavenging activities increased from 8.04% to 46.33% and from 17.02% to 36.43%, respectively, while ferric reducing antioxidant power (FRAP) increased from 6.17 to 23.64 mM FeSO_4_ equivalents/g. Total polyphenol content (TPC) increased from 0.66 to 1.46 mg gallic acid equivalents/g, and total flavonoid content (TFC) increased from 0.20 to 0.85 mg catechin equivalents/g. These results show that increasing CTP raised the extractable phenolic indices, radical scavenging activity, and reducing capacity measured in the baked samples. The findings establish a formulation-dependent antioxidant response in the final baked products and provide a basis for future compound-specific studies of phenolic retention during processing.

## 1. Introduction

Phenolic compounds are widely studied natural bioactive constituents because they can take part in antioxidant and redox reactions [1]. Their activity is mainly related to electron donation, hydrogen transfer, and radical scavenging, although the measured response can vary with the assay conditions and chemical environment. During heating, phenolic compounds may undergo oxidation, degradation, polymerization, or interactions with other matrix components, which can change the apparent antioxidant activity [1].

Baked systems provide a useful thermal processing matrix because heating involves water loss, protein denaturation, starch transformation, and nonenzymatic browning reactions, including the Maillard reaction. These changes affect texture and color and also influence the chemical environment surrounding phenolic compounds. Recent studies of cookies enriched with plant powders or extracts have shown that the formulation can influence hardness, color, phenolic indices, and antioxidant activity [2,3,4,5,6,7,8,9,10]. Melanoidin-rich extracts from baked cookies have also shown radical scavenging activity, illustrating a potential contribution from Maillard reaction products [11]. A cookie matrix can therefore be used to evaluate the antioxidant response of a plant ingredient after baking.

*Cynanchum thesioides* (Freyn) K. Schum is an edible and medicinal plant reported to contain flavonoids, triterpenes, and other phenolic constituents. In our previous study, the methanol extract yield from material collected in Inner Mongolia was 13.33%, and the extract and its solvent fractions showed measurable total polyphenol content and antioxidant activity [12]. The ethyl acetate and n-butanol fractions contained 112.54 and 100.86 mg gallic acid equivalents/g, respectively [12]. These extract-based findings establish the presence of solvent-extractable antioxidant constituents in this botanical material, although their behavior after incorporation into a baked cereal matrix remains unclear.

In this study, dried *Cynanchum thesioides* powder was incorporated into a cookie matrix as a thermal processing model. The objective was to evaluate physicochemical properties, phenolic indices, and antioxidant activity after baking. Moisture content, spread ratio, density, hardness, color, pH, and soluble solids were measured to characterize matrix changes. Total polyphenol content (TPC), total flavonoid content (TFC), 2,2-diphenyl-1-picrylhydrazyl (DPPH) and 2,2′-azino-bis(3-ethylbenzothiazoline-6-sulfonic acid) (ABTS) radical scavenging activities, and ferric reducing antioxidant power (FRAP) were measured in the baked samples. The present work focuses on formulation-level responses in the final baked products, providing a foundation for future compositional profiling and direct evaluation of compound-specific retention before and after baking.

## 2. Results and Discussion

### 2.1. Physicochemical Changes in the Baked Matrix After CTP Incorporation

The incorporation of dried *Cynanchum thesioides* powder (CTP) changed several properties of the baked matrix. As shown in Table 1, Table 2 and Table 3, increasing the CTP level affected moisture content, spread ratio, hardness, color, pH, and soluble solids, while density did not differ significantly among samples. These coordinated changes indicate that partial replacement of wheat flour with CTP modified both dough composition and structural development during baking.

#### 2.1.1. Moisture Content, Spread Ratio, Density, and Hardness

CTP significantly affected moisture content, spread ratio, and hardness, whereas density was unchanged (Table 1). The progressive decrease in moisture content is consistent with formulation-dependent differences in water retention and loss during baking. Partial flour replacement, changes in dough spreading and exposed surface area, and differences in the balance of fiber, protein, and low-molecular-weight solids may all contribute. Recent cookie studies likewise show that plant powders can either increase or decrease final moisture depending on their composition and processing history [3,7,9]. Together, these factors explain the formulation-level trend and identify water-holding capacity and proximate composition as priorities for ingredient characterization.

The spread ratio increased slightly as the CTP level increased. Samples containing 3% and 5% CTP showed significantly higher spread ratios than the control. This pattern is consistent with partial dilution of gluten proteins and a less continuous starch and protein network, which can permit greater lateral expansion during baking. Interactions among phenolic compounds, proteins, and starch through hydrogen bonding or hydrophobic forces may also modify dough viscosity. Density did not differ significantly among samples, indicating that the overall mass-to-volume relationship was not strongly affected by CTP addition.

Hardness decreased by approximately 45% between the control and 5% CTP formulations. This result is consistent with weakening of the load-bearing gluten–starch network when flour is replaced and particulate material interferes with network continuity. Phenolic–protein and phenolic–polysaccharide interactions may further modify protein association and starch organization through hydrogen bonding and hydrophobic interactions. Recent studies show that fiber-rich plant powders may either soften or harden cookies depending on fiber solubility, particle size, and water binding [3,7,8,9,10]. Together, network dilution and particulate interference provide a formulation-level explanation consistent with the observed softening.

#### 2.1.2. Color Characteristics

The color values of the baked matrix were significantly affected by CTP incorporation, as shown in Table 2. Lightness (L*) decreased from 78.24 in the control sample to 51.48 at 5% CTP, indicating progressive darkening with increasing CTP level. Redness (a*) increased from 4.81 to 10.07, while yellowness (b*) decreased from 37.16 to 29.56. The change likely reflects the combined effects of natural CTP pigments, phenolic oxidation, and Maillard or caramelization products. Similar changes have been reported in cookies enriched with plant powders or extracts [2,3,4,5,6,10].

**Figure 1:**
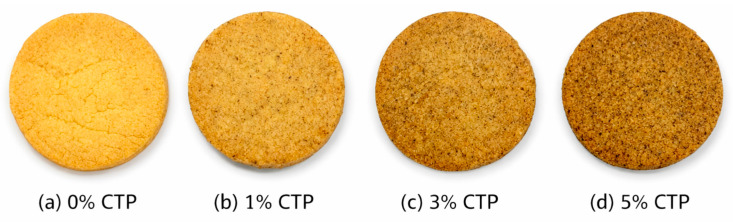
Appearance of baked samples containing different levels of dried *Cynanchum thesioides* powder (CTP) at 0%, 1%, 3%, and 5% (*w*/*w*).

The gradual darkening visible in Figure 1 agrees with the decrease in L* and the accompanying shifts in a* and b*. Together, the photographs and instrumental measurements show a coherent concentration-dependent color response to CTP incorporation.

#### 2.1.3. pH and Soluble Solids (°Brix)

The small but significant decrease in cookie-extract pH with increasing CTP indicates that the formulation altered the acid–base environment of the baked matrix. Organic acids and phenolic hydroxyl groups in CTP, together with formulation-dependent extraction after baking, are plausible contributors. The observed pH response also identifies powder pH and titratable acidity as focused targets for ingredient characterization.

The slight increase in soluble solids may reflect extractable sugars, acids, minerals, or other water-soluble constituents introduced by CTP, together with formulation-dependent extraction after baking. The narrow absolute range indicates a modest effect; accordingly, °Brix is interpreted here as an overall measure of water-extractable solids in the baked sample extracts.

#### 2.1.4. Integrated Interpretation of Matrix Changes

Taken together, the higher spread ratio and lower hardness suggest disruption or dilution of the gluten–starch network, whereas the color and pH shifts indicate simultaneous changes in the chemical environment during baking. Hydrogen bonding and hydrophobic association among phenolic constituents, proteins, and starch provide plausible molecular pathways linking CTP addition with matrix structure. Together, these interactions provide a coherent framework for the matrix response.

These physical changes may also affect solvent access during post-baking extraction. A less rigid matrix could expose entrapped constituents, while phenolic binding to proteins or polysaccharides could reduce their extractability. The measured response therefore integrates CTP addition, thermal transformation, matrix binding, and extraction efficiency.

### 2.2. Phenolic Content and Antioxidant Activity After Thermal Processing

The antioxidant activity and phenolic indices of the baked samples are presented in Table 4, Table 5 and Table 6. Increasing the CTP level increased radical scavenging activity, reducing capacity, TPC, and TFC. The complementary assays characterize radical scavenging under different reaction conditions (DPPH and ABTS), electron-transfer capacity in an acidic medium (FRAP), and colorimetric phenolic and flavonoid indices (TPC and TFC) [13,14,15].

#### 2.2.1. DPPH and ABTS Radical Scavenging Activity

The radical scavenging activity of the baked samples was evaluated using DPPH and ABTS assays, and the results are shown in Table 4. Both assays increased with CTP level, although they use radicals with different solubility and reaction conditions.

DPPH radical scavenging activity increased from 8.04% in the control sample to 46.33% at 5% CTP. This increase shows greater radical scavenging activity in the final extracts. DPPH mainly responds to compounds able to donate hydrogen atoms or electrons under the assay conditions [16]. Similar increases have been reported in cookies enriched with plant powders or extracts [2,4,5,6,7,8,9,10].

ABTS radical scavenging activity increased from 17.02% in the control sample to 36.43% at 5% CTP. The ABTS assay can respond to antioxidants in a different reaction environment from DPPH [14,17]. The concordant concentration-dependent responses in DPPH and ABTS provide complementary evidence that CTP incorporation enhanced radical scavenging across two assay environments.

#### 2.2.2. Ferric Reducing Antioxidant Power

Ferric reducing antioxidant power (FRAP) was measured to evaluate the reducing capacity of the baked samples. As shown in Table 5, FRAP increased from 6.17 mM FeSO_4_ equivalents/g in the control sample to 23.64 mM FeSO_4_ equivalents/g at 5% CTP.

FRAP reflects the ability of compounds in the extract to reduce ferric ions under acidic conditions [18]. CTP may supply phenolic and other reducing constituents, while baking at 180 °C may also generate reducing Maillard reaction products, including melanoidins with reported antioxidant activity in cookie extracts [11]. Both pathways are chemically plausible, and the measured FRAP response represents their net contribution in the final extracts. These complementary pathways provide a focused basis for comparative profiling of unbaked and baked matrices with Maillard reaction markers.

#### 2.2.3. Total Polyphenol and Total Flavonoid Content

The total polyphenol content (TPC) and total flavonoid content (TFC) of the baked samples are presented in Table 6. Both increased significantly with CTP level. TPC increased from 0.66 mg GAE/g in the control sample to 1.46 mg GAE/g at 5% CTP, while TFC increased from 0.20 mg CE/g to 0.85 mg CE/g over the same range. Similar increases have been reported in recent studies of cookies enriched with plant materials [2,3,4,5,6,7,8,9,10].

The parallel increases in TPC, TFC, DPPH, ABTS, and FRAP are consistent with a contribution from extractable phenolic constituents. The Folin–Ciocalteu reagent can also respond to nonphenolic reducing substances, and the aluminum chloride assay has compound-dependent response factors [19,20]; baking products may likewise contribute to reducing assays. The data are therefore interpreted as an integrated increase in phenolic indices and antioxidant response.

Compound-resolved profiling before and after baking would extend these findings by identifying the contributing constituents and quantifying formulation-dependent retention, transformation, and extractability.

#### 2.2.4. Formulation Significance and Future Directions

The coherent dose–response across five analytical outcomes demonstrates that CTP supplementation increased the extractable antioxidant response of the final cookie matrix. Even at replacement levels up to 5%, CTP raised phenolic indices and in vitro antioxidant capacity while producing systematic changes in texture and color. This combination supports its potential as a functional ingredient for antioxidant-enriched baked formulations.

For consumer-oriented development, the next step is to determine how the chemical response translates into sensory acceptance and gastrointestinal bioaccessibility, together with confirmation of ingredient safety under intended use conditions. These evaluations would define the practical nutritional relevance of the CTP-enriched formulations.

### 2.3. Relationship Between Matrix Changes and Antioxidant Response

Increasing CTP simultaneously altered the physical matrix and increased all measured antioxidant indices. A less continuous gluten–starch network may improve solvent access to entrapped constituents, whereas phenolic–macromolecule binding may moderate extractability. The observed response therefore reflects the combined influence of CTP level, baking reactions, matrix interactions, and analytical extraction.

**Figure 2:**
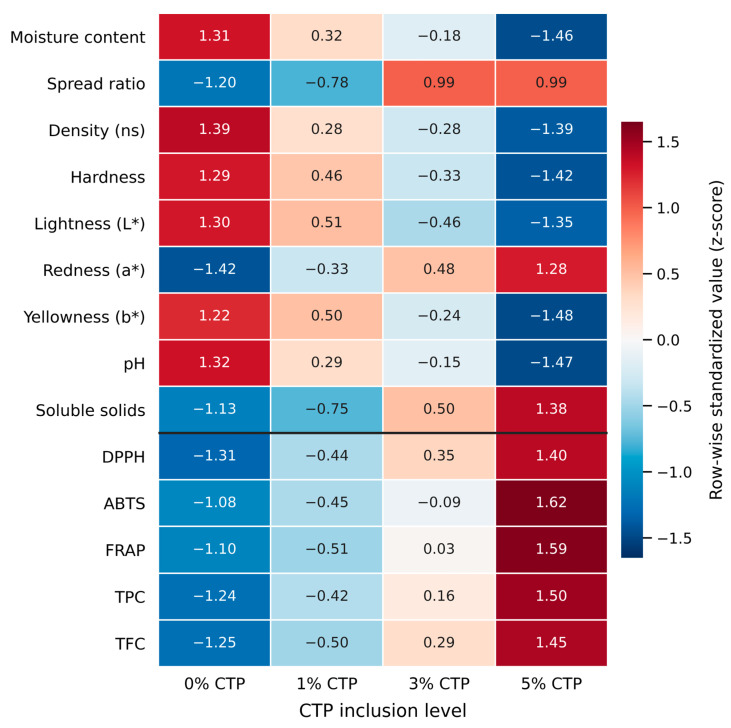
Descriptive heatmap of row-wise standardized treatment means at each CTP concentration. Colors show the direction of change within each variable and do not compare effect magnitudes across variables. L*, a*, and b* denote lightness, red–green chromaticity, and yellow–blue chromaticity in the CIELAB color space, respectively; the asterisks are part of the CIELAB notation and do not indicate statistical significance. Density did not differ significantly among formulations (*p* ≥ 0.05).

Figure 2 summarizes these coordinated formulation-level trends using a row-wise standardized heatmap of the treatment means. Increasing CTP was descriptively associated with lower moisture, hardness, L*, b*, and pH and with higher spread ratio, a*, soluble solids, DPPH, ABTS, FRAP, TPC, and TFC; the small density change was not significant. Because each variable was standardized separately, the colors show direction within each variable rather than effect magnitude across variables. The visualization highlights the overall response pattern and provides a basis for correlation analysis in future studies with independent baking batches.

## 3. Materials and Methods

### 3.1. Materials

Wheat flour, butter, sugar, salt, and eggs used for preparation of the baked matrix were purchased from a local commercial supplier in Daejeon, Republic of Korea, and used without further treatment.

Dried *Cynanchum thesioides* (Freyn) K. Schum was purchased from a local herbal market in Jiuyuan District, Baotou, Inner Mongolia Autonomous Region, China. The material was obtained in an air-dried form, ground, passed through a 100-mesh sieve, and stored in airtight containers protected from light until use. Our previous study of comparable *C. thesioides* stems, leaves, and flower buds from Inner Mongolia documented solvent-extractable phenolic constituents and antioxidant activity after methanol extraction and sequential fractionation [12]. These findings provide botanical and bioactivity context for the present formulation study.

All reagents used for antioxidant assays, including 2,2-diphenyl-1-picrylhydrazyl (DPPH), 2,2′-azino-bis(3-ethylbenzothiazoline-6-sulfonic acid) (ABTS), Folin–Ciocalteu reagent, 2,4,6-tris(2-pyridyl)-s-triazine (TPTZ), ferric chloride (FeCl_3_), and other reagents required for the ferric reducing antioxidant power (FRAP) assay, were of analytical grade and purchased from Sigma-Aldrich (St. Louis, MO, USA).

### 3.2. Preparation of Baked Samples Containing Cynanchum thesioides Powder

Baked samples were prepared by partially replacing wheat flour with CTP at 0%, 1%, 3%, and 5% (*w*/*w*). The formulation of each sample is shown in Table 7.

Butter and sugar were mixed for approximately 3 min until a uniform cream was obtained. Eggs were then added and mixed thoroughly. Wheat flour, salt, and the designated amount of CTP were blended separately and gradually added to the wet mixture to form a homogeneous dough.

The dough was sheeted to a thickness of approximately 5 mm and cut into circular pieces with a diameter of 40 mm using a standard mold. The shaped dough pieces were baked in a preheated electric deck oven (FDO-7102, Daeyung Bakery Machinery Ind. Co., Ltd., Seoul, Republic of Korea) at 180 °C for 15 min. After baking, the samples were cooled at room temperature for 30 min and stored in sealed polyethylene containers at ambient temperature until analysis.

### 3.3. Measurement of Physicochemical Properties

Physicochemical properties were measured to evaluate changes in the baked matrix resulting from CTP incorporation. The measured parameters included moisture content, spread ratio, density, hardness, color values (L*, a*, and b*), pH, and soluble solids (°Brix). The number and type of measurements are specified below. Each formulation was produced in one baking trial.

#### 3.3.1. Moisture Content

Moisture content was determined using a hot-air drying oven (SW-90D, Sang Woo Scientific Co., Ltd., Bucheon, Republic of Korea). Approximately 3 g of finely ground sample was weighed into a previously dried aluminum dish and dried at 105 °C for 24 h or until a constant weight was obtained. The samples were cooled in a desiccator before weighing. Moisture content was calculated based on weight loss and expressed as a percentage. Three analytical determinations were performed for each formulation (*n* = 3).

#### 3.3.2. Spread Ratio and Density

The diameter and thickness of the baked samples were measured using a digital caliper (CD-15APX, Mitutoyo Corporation, Kawasaki, Japan). For each formulation, five cookie units were randomly selected and measured (*n* = 5), and the mean values were used for calculation. The spread ratio was calculated as the ratio of diameter to thickness.

Density was calculated as the ratio of sample mass to volume. Three cookie units were measured for each formulation (*n* = 3). The volume of each sample was estimated by assuming a cylindrical shape, using Equation (1):

(1) $$V=\pi {\left(\frac{d}{2}\right)}^{2}h$$

where V is the estimated sample volume, d is the measured diameter, and h is the measured thickness. Diameter and thickness values were converted to centimeters before calculation, and density was expressed as g/mL.

#### 3.3.3. Texture Analysis

Hardness was measured using a texture analyzer (TA-XT2i, Stable Micro Systems Ltd., Godalming, Surrey, UK) equipped with a cylindrical compression probe with a diameter of 36 mm. Each sample was positioned at the center of the probe and compressed at a test speed of 1.0 mm/s until fracture occurred. The maximum force recorded during compression was defined as hardness and expressed in grams (g). Five cookie units were tested for each formulation (*n* = 5).

#### 3.3.4. Color Measurement

Color values (L*, a*, and b*) were measured using a colorimeter (CR-400, Konica Minolta, Osaka, Japan) calibrated with a standard white calibration plate before measurement. Five cookies were evaluated for each formulation (*n* = 5). Three surface readings were taken from each cookie and averaged to give one value per cookie.

#### 3.3.5. pH and Soluble Solids (°Brix)

For pH measurement, 5 g of ground sample was mixed with 45 mL of distilled water and stirred at room temperature for 5 min to obtain a uniform dispersion. The suspension was filtered before measurement. The pH was measured at room temperature using a calibrated pH meter (Orion Star A211, Thermo Scientific, Waltham, MA, USA).

Soluble solids (°Brix) were determined using a digital refractometer (PAL-1, Atago Co., Ltd., Tokyo, Japan) after filtration of the aqueous extract. Both pH and soluble solids were measured in three analytical determinations for each formulation (*n* = 3).

### 3.4. Antioxidant Activity Assays

Antioxidant activity of the baked sample extracts was evaluated using DPPH, ABTS, and FRAP assays. These assays were selected to assess radical scavenging activity and reducing capacity under different reaction conditions [13,14,15].

For extract preparation, ground samples (10 g) were mixed with 100 mL of 70% ethanol, giving a 1:10 ratio of sample to solvent (*w*/*v*). Extraction was carried out at room temperature for 12 h under continuous shaking at 150 rpm using an orbital shaker (SHO-1D, Daihan Scientific Co., Ltd., Seoul, Republic of Korea). The mixtures were then centrifuged at 1811× *g* for 10 min using a refrigerated centrifuge (Mega 17R, Hanil Scientific Inc., Gimpo, Republic of Korea). The supernatants were collected and filtered before analysis. The extracts were used for DPPH, ABTS, and FRAP assays as described below. Three analytical determinations were performed for each formulation (*n* = 3).

#### 3.4.1. DPPH Radical Scavenging Activity

DPPH radical scavenging activity was determined according to the method described by Blois, with minor modifications [16]. Briefly, 1 mL of sample extract was mixed with 1 mL of DPPH solution (0.2 mM in ethanol). The mixture was allowed to react in the dark at room temperature for 30 min. The absorbance was then measured at 517 nm using a UV-Vis spectrophotometer (Optizen 2020 UV Plus, Mecasys Co., Ltd., Daejeon, Republic of Korea).

#### 3.4.2. ABTS Radical Scavenging Activity

ABTS radical scavenging activity was determined according to a modified method described by Re et al. [17]. The ABTS radical cation was generated by reacting an aqueous ABTS solution with potassium persulfate and allowing the mixture to stand in the dark at room temperature for 14–16 h. Before analysis, the ABTS radical solution was diluted with ethanol to obtain an absorbance of 0.70 ± 0.02 at 734 nm.

For the assay, 50 μL of sample extract was mixed with 1.0 mL of the diluted ABTS radical solution and allowed to react in the dark for 2.5 min. The absorbance was then measured at 734 nm using a UV-Vis spectrophotometer.

For both DPPH and ABTS assays, radical scavenging activity was calculated using Equation (2):

(2) $$\text{Radical scavenging activity}\left(\%\right)=\left(1-\frac{A\_sample}{A\_control}\right)\times 100$$

where A_control is the absorbance of the control solution and A_sample is the absorbance of the sample extract.

#### 3.4.3. Ferric Reducing Antioxidant Power

Ferric reducing antioxidant power (FRAP) was determined according to the method described by Benzie and Strain, with minor modifications [18]. The FRAP reagent was freshly prepared by mixing acetate buffer (300 mM, pH 3.6), TPTZ solution (10 mM in 40 mM HCl), and ferric chloride solution (20 mM FeCl_3_·6H_2_O) at a ratio of 10:1:1 (*v*/*v*/*v*).

For the assay, 30 μL of sample extract was mixed with 90 μL of distilled water and 0.9 mL of FRAP reagent. The reaction mixture was incubated at 37 °C for 10 min, and the absorbance was measured at 593 nm using a UV-Vis spectrophotometer.

FRAP values were calculated using a calibration curve prepared with ferrous sulfate (FeSO_4_·7H_2_O) and expressed as mM FeSO_4_ equivalents per gram of sample.

### 3.5. Determination of Total Polyphenol Content and Total Flavonoid Content

The total polyphenol content (TPC) was determined using the Folin–Ciocalteu method described by Singleton et al., with minor modifications [19]. The sample extract was mixed with diluted Folin–Ciocalteu reagent and allowed to react briefly before the addition of sodium carbonate solution. The reaction mixture was then incubated at room temperature for 1 h, and the absorbance was measured at 765 nm using a UV-Vis spectrophotometer. Gallic acid was used as the standard compound for calibration, and the results were expressed as milligrams of gallic acid equivalents per gram of sample (mg GAE/g).

The total flavonoid content (TFC) was determined using the aluminum chloride colorimetric method described by Jia et al. [20]. The sample extract was sequentially reacted with sodium nitrite, aluminum chloride, and sodium hydroxide solutions, and the absorbance was measured at 510 nm using a UV-Vis spectrophotometer. Catechin was used as the standard compound for calibration, and the results were expressed as milligrams of catechin equivalents per gram of sample (mg CE/g).

TPC and TFC were each measured in three analytical determinations for every formulation (*n* = 3), and all calibration curves showed good linearity (R^2^ > 0.99).

### 3.6. Statistical Analysis

Each cookie formulation was prepared in a single baking trial. Within this trial, five individual cookie units were evaluated for spread ratio, hardness, and color (*n* = 5), while three cookie units were evaluated for density (*n* = 3). Moisture content, pH, soluble solids, DPPH, ABTS, FRAP, TPC, and TFC were each measured in three analytical determinations (*n* = 3). For color analysis, three surface readings were averaged for each cookie unit. Results are expressed as mean ± standard deviation (SD). One-way analysis of variance (ANOVA), followed by Duncan’s multiple range test, was performed using IBM SPSS Statistics version 26.0 (IBM Corp., Armonk, NY, USA), with differences considered significant at *p* < 0.05. These analyses characterize formulation differences within the single baking trial, while reproducibility at the production level will be evaluated using independent baking trials. The descriptive heatmap was generated using Matplotlib version 3.10.8 (Matplotlib Development Team).

## 4. Conclusions

This study showed that dried *Cynanchum thesioides* powder changed moisture content, spread ratio, hardness, color, pH, and soluble solids in a baked matrix, while density was unchanged. DPPH, ABTS, FRAP, TPC, and TFC increased with CTP concentration, demonstrating a stronger extractable antioxidant response in the final baked samples. These coordinated responses establish CTP concentration as a formulation factor that simultaneously modifies matrix properties and antioxidant indices in the final baked product.

The lower hardness is consistent with disruption of the gluten and starch network, while the antioxidant response may reflect both constituents introduced by CTP and reducing products formed during baking. Accordingly, the chemical and textural trends observed here describe the structural behavior of the formulations tested within the present baking trial, while independent baking batches are needed to establish reproducibility during production. Direct CTP characterization and analysis of individual compounds before and after baking will further clarify the mechanism. Sensory acceptance and bioaccessibility assessments will help determine the practical potential of CTP in food products.

## Figures and Tables

**Table 1:** Moisture content, spread ratio, density, and hardness of baked samples containing different levels of dried *Cynanchum thesioides* powder (CTP).

	CTP			
	0%	1%	3%	5%
Moisture content (%)	4.78 ± 0.02 ^a^	4.17 ± 0.22 ^b^	3.86 ± 0.13 ^c^	3.07 ± 0.04 ^d^
Spread ratio	9.01 ± 0.19 ^b^	9.15 ± 0.27 ^b^	9.73 ± 0.15 ^a^	9.73 ± 0.15 ^a^
Density (g/mL)	1.07 ± 0.03 ^a^	1.05 ± 0.01 ^a^	1.04 ± 0.03 ^a^	1.02 ± 0.03 ^a^
Hardness (g)	1328.74 ± 0.81 ^a^	1145.98 ± 0.78 ^b^	971.46 ± 0.66 ^c^	729.94 ± 0.84 ^d^

CTP was added at 0%, 1%, 3%, and 5% (*w*/*w*) based on wheat flour weight. Values are expressed as mean ± SD from the single baking trial (moisture content, three analytical determinations; spread ratio and hardness, five cookie units; density, three cookie units). Entries shown as mean (SD ± 0.01) indicate SD values recorded as 0.00 in the archived two-decimal summary. Means within the same row not sharing a common letter are significantly different (*p* < 0.05) according to Duncan’s multiple range test.

**Table 2:** Color values of baked samples containing different levels of dried *Cynanchum thesioides* powder (CTP).

Color Values	CTP			
	0%	1%	3%	5%
Lightness (L*)	78.24 ± 0.04 ^a^	70.33 ± 0.01 ^b^	60.50 ± 0.02 ^c^	51.48 ± 0.02 ^d^
Redness (a*)	4.81 ± 0.02 ^d^	6.94 ± 0.02 ^c^	8.51 ± 0.01 ^b^	10.07 ± 0.01 ^a^
Yellowness (b*)	37.16 ± 0.02 ^a^	35.14 ± 0.01 ^b^	33.04 ± 0.01 ^c^	29.56 ± 0.02 ^d^

CTP was added at 0%, 1%, 3%, and 5% (*w*/*w*) based on wheat flour weight. Values are expressed as mean ± SD for five cookie units from the single baking trial (*n* = 5); three surface readings were averaged for each cookie. The entry shown as mean (SD ± 0.01) indicates an SD recorded as 0.00 in the archived two-decimal summary. Means within the same row not sharing a common letter are significantly different (*p* < 0.05) according to Duncan’s multiple range test.

**Table 3:** pH and soluble solids of baked samples containing different levels of dried *Cynanchum thesioides* powder (CTP).

	CTP			
	0%	1%	3%	5%
pH	5.90 ± 0.01 ^a^	5.83 ± 0.01 ^b^	5.80 ± 0.01 ^c^	5.71 ± 0.01 ^d^
Soluble solids (°Brix)	2.50 ± 0.01 ^c^	2.53 ± 0.06 ^bc^	2.63 ± 0.06 ^ab^	2.70 ± 0.10 ^a^

CTP was added at 0%, 1%, 3%, and 5% (*w*/*w*) based on wheat flour weight. Values are expressed as mean ± SD for three analytical determinations within the single baking trial (*n* = 3). Entries shown as mean (SD ± 0.01) indicate SD values recorded as 0.00 in the archived two-decimal summary. Means within the same row not sharing a common letter are significantly different (*p* < 0.05) according to Duncan’s multiple range test.

**Table 4:** DPPH and ABTS radical scavenging activity of baked samples containing different levels of dried *Cynanchum thesioides* powder (CTP).

	CTP			
	0%	1%	3%	5%
DPPH radical scavenging activity (%)	8.04 ± 0.11 ^d^	20.36 ± 0.51 ^c^	31.59 ± 0.22 ^b^	46.33 ± 0.11 ^a^
ABTS radical scavenging activity (%)	17.02 ± 0.49 ^d^	21.57 ± 0.27 ^c^	24.19 ± 0.13 ^b^	36.43 ± 0.55 ^a^

CTP was added at 0%, 1%, 3%, and 5% (*w*/*w*) based on wheat flour weight. Values are expressed as mean ± SD for three analytical determinations within the single baking trial (*n* = 3). Means within the same row not sharing a common letter are significantly different (*p* < 0.05) according to Duncan’s multiple range test.

**Table 5:** Ferric reducing antioxidant power (FRAP) of baked samples containing different levels of dried *Cynanchum thesioides* powder (CTP).

	CTP			
	0%	1%	3%	5%
FRAP (mM FeSO_4_ equivalents/g)	6.17 ± 0.07 ^d^	10.01 ± 0.14 ^c^	13.52 ± 0.12 ^b^	23.64 ± 0.70 ^a^

CTP was added at 0%, 1%, 3%, and 5% (*w*/*w*) based on wheat flour weight. Values are expressed as mean ± SD for three analytical determinations within the single baking trial (*n* = 3). Means within the same row not sharing a common letter are significantly different (*p* < 0.05) according to Duncan’s multiple range test.

**Table 6:** Total polyphenol content and total flavonoid content of baked samples containing different levels of dried *Cynanchum thesioides* powder (CTP).

	CTP			
	0%	1%	3%	5%
Total polyphenol (mg GAE/g)	0.66 ± 0.01 ^d^	0.90 ± 0.02 ^c^	1.07 ± 0.02 ^b^	1.46 ± 0.03 ^a^
Total flavonoid (mg CE/g)	0.20 ± 0.02 ^d^	0.38 ± 0.01 ^c^	0.57 ± 0.01 ^b^	0.85 ± 0.01 ^a^

CTP was added at 0%, 1%, 3%, and 5% (*w*/*w*) based on wheat flour weight. Values are expressed as mean ± SD for three analytical determinations within the single baking trial (*n* = 3). Means within the same row not sharing a common letter are significantly different (*p* < 0.05) according to Duncan’s multiple range test.

**Table 7:** Formulation of baked samples containing different levels of dried *Cynanchum thesioides* powder (CTP).

Ingredients (g)	CTP (%, *w*/*w*)			
	0%	1%	3%	5%
Flour	100	99	97	95
CTP	0	1	3	5
Butter	60	60	60	60
Sugar	40	40	40	40
Salt	1	1	1	1
Egg	20	20	20	20

CTP was incorporated at 0%, 1%, 3%, and 5% (*w*/*w*) by replacing wheat flour on a total flour basis of 100 g.

## Data Availability

The data presented in this study are available within the article.
